# Distribution and Genetic Variability of *Fusarium oxysporum* Associated with Tomato Diseases in Algeria and a Biocontrol Strategy with Indigenous *Trichoderma* spp.

**DOI:** 10.3389/fmicb.2018.00282

**Published:** 2018-02-21

**Authors:** Ali Debbi, Houda Boureghda, Enrique Monte, Rosa Hermosa

**Affiliations:** ^1^Laboratory of Phytopathology and Molecular Biology, Department of Botany, National Superior School of Agronomy, Algiers, Algeria; ^2^Laboratory of Mycology, Center of Biotechnology Research, Constantine, Algeria; ^3^Department of Microbiology and Genetics, Spanish-Portuguese Institute for Agricultural Research (CIALE), University of Salamanca, Salamanca, Spain

**Keywords:** ISSR, antagonism, antibiosis, biological control, *Trichoderma ghanense*, *Fusarium oxysporum* f. sp. *radicis lycopersici*, *Fusarium oxysporum* f. sp. *lycopersici*

## Abstract

Fifty fungal isolates were sampled from diseased tomato plants as result of a survey conducted in seven tomato crop areas in Algeria from 2012 to 2015. Morphological criteria and PCR-based identification, using the primers PF02 and PF03, assigned 29 out of 50 isolates to *Fusarium oxysporum* (*Fo*). The banding patterns amplified for genes *SIX1, SIX3* and *SIX4* served to identify races 2 and 3 of *Fo* f. sp. *lycopersici* (FOL), and *Fo* f. sp. *radicis lycopersici* (FORL) among the Algerian isolates. All FOL isolates showed pathogenicity on the susceptible tomato cv. “Super Marmande,” while nine of out 10 Algerian FORL isolates were pathogenic on tomato cv. “Rio Grande.” Inter simple sequence repeat (ISSR) fingerprints showed high genetic diversity among Algerian *Fo* isolates. Seventeen Algerian *Trichoderma* isolates were also obtained and assigned to the species *T. asperellum* (12 isolates), *T. harzianum* (four isolates) and *T. ghanense* (one isolate) based on ITS and *tef1*α gene sequences. Different *in vitro* tests identified the antagonistic potential of native *Trichoderma* isolates against FORL and FOL. Greenhouse biocontrol assays performed on “SM” tomato plants with *T. ghanense* T8 and *T. asperellum* T9 and T17, and three *Fo* isolates showed that isolate T8 performed well against FORL and FOL. This finding was based on an incidence reduction of crown and root rot and Fusarium wilt diseases by 53.1 and 48.3%, respectively.

## Introduction

*Fusarium oxysporum* Schlecht (*Fo*) is a free-living ascomycete fungus with no known sexual state. *Fo* is a complex species comprised of ubiquitous soil-borne plant pathogens, with ca. 120 *formae speciales* (ff. spp.) based on host specificity (Michielse and Rep, 2009; Arie, 2010). The different ff. spp. show considerable genetic diversity and have polyphyletic origin (O'Donnell et al., 1998; Nirmaladevi et al., 2016). *Fo* causes significant economic losses of many crops including tomato (*Solanum lycopersium* L.), which is one of the most worldwide cultivated vegetable crops. *Fo* diseases in tomato are mainly caused by f. sp. *radicis-lycopersici* Jarvis and Shomaker (FORL), responsible for crown and root rot, and by f. sp. *lycopersici* (Sacc.) Snyder and Hansen (FOL), responsible for vascular wilt disease (Edel-Hermann et al., 2012), and although both ff. spp. infect the same host plant, FOL and FORL have strict host specificity. Three physiological races of FOL (1, 2, and 3) have been differentiated depending on their ability to infect tomato cultivars carrying different resistance *loci* (Mes et al., 1999). The use of resistant cultivars and resistant rootstocks remains the most appropriate way to prevent *Fo* diseases in tomato production. Hence, determining which pathogens are emerging in the field is important in order to select the most suitable tomato cultivar. Since pathogenic strains of *Fo* cannot be identified morphologically, pathogenicity tests are commonly used on different tomato cultivars. However, these methods are very time-consuming and expensive (Baysal et al., 2009), and also the results of these types of biological tests can be affected by variations in temperature (Boix-Ruíz et al., 2015).

The gene sequence variability found in polygalacturonases, major enzymes involved in *Fo*-plant interactions (Di Pietro and Roncero, 1998), has been useful to study the genetic diversity in populations of this fungus (Kawabe et al., 2005). A PCR-based technique, using a set of primers specific to the sequences of the endo-polygalacturonase gene *pg1* and the exo-polygalacturonase gene *pgx4* of *Fo* isolates from Japan, allowed FOL and FORL, and the races of FOL (Hirano and Arie, 2006) to be differentiated. However, discrepancies have been observed in identifying some isolates collected from tomato crops in other areas of the world, such as those from the Mediterranean coast of Turkey, with pathogenicity tests and PCR carried out using this set of primers (Baysal et al., 2009; Çolak and Biçici, 2013).

FOL isolates appear to have horizontally transferred accessory chromosomes (Ma, 2014) which encode a number of putative effectors, including the set of the secreted in xylem (SIX) proteins (Houterman et al., 2009). Several SIX genes have been associated with the three races of FOL, and the molecular markers developed for these genes provide a robust PCR-based method for identifying the host specificity of FOL isolated from plant tissues (Lievens et al., 2009; Jelinski et al., 2017). In addition, the presence of *SIX1* can be used to identify FOL isolates. Moreover, *SIX4* allows the identification of race 1 isolates, and *SIX3* variations can serve to differentiate race 2 from race 3 isolates (Lievens et al., 2009). A previous study exploring the genetic diversity of *Fo* and other *Fusarium* spp. pathogenic on tomato in different Mediterranean countries, which combine the use of the presence of *SIX1*, intergenic spacer (IGS) DNA typing and vegetative compatibility grouping (VCG), identified 27 out of 27 *Fo* Algerian isolates as FORL (Edel-Hermann et al., 2012). Unfortunately, cultivars of tomato with resistance to FORL are not yet commercially available. And, crown and root rot disease caused by FORL is widely present in most of the African and Asian Mediterranean countries, including Algeria, where tomato production is economically important, occupying second place after potato. Moreover, there are no reliable data regarding Fusarium wilt, caused by FOL races, in Algerian tomato cultivation areas.

*Trichoderma* species (teleomorph *Hypocrea*) are cosmopolitan filamentous fungi frequently found in agricultural habitats because of their ability to colonize the rhizosphere and progress in different soils (Hermosa et al., 2004; Rubio et al., 2005). The biocontrol capability of these fungi is well recognized since they are antagonists of phytopathogenic fungi, oomycetes and nematodes (Lorito et al., 2010; Medeiros et al., 2017). The biocontrol mechanisms of *Trichoderma* are at least based on competition for nutrients, the production of hydrolytic enzymes and/or antibiotics (Harman et al., 2004). In addition, a systemic activation of plant defense responses against biotic and abiotic damages has been observed for selected rhizosphere-competent *Trichoderma* strains (Hermosa et al., 2012; Ruocco et al., 2015; Rubio et al., 2017a), and the ability of *Trichoderma* spp. to reduce Fusarium wilt in tomato has been previously described (Cotxarrera et al., 2002; Taghdi et al., 2015).

The aim of the present study was to identify the *Fo* pathogenic types present in the current seven major tomato-growing areas in Algeria, to explore their genetic diversity and to propose environmentally friendly practices for controlling *Fo* diseases in tomato. To this end, a collection of *Trichoderma* spp. isolates was obtained from soils sampled in tomato plantations with low incidence of Fusarium wilt and from healthy nursery tomato plants in Algeria. These isolates were then molecularly identified and screened using both *in vitro* and *in vivo* assays to explore biocontrol potentialities against FOL and FORL. In addition, we wanted to contribute to previous studies on *Trichoderma* diversity in the North African Mediterranean countries in which *T. atroviride* and *T. hamatum* were prevalent in cultivated soils from Tunisia (Sadfi-Zouaoui et al., 2009), and *T. asperellum, T. hamatum* and *T. virens* were found in soil and compost samples in Morocco (Taghdi et al., 2015). It is noteworthy that race 2 and 3 of FOL are being reported for the first time in Algeria and that as far as we know *T. ghanense* has not been previously recorded in the Mediterranean North African countries.

## Materials and methods

### Collection of plant and soil samples and fungal isolation

**Figure 1:**
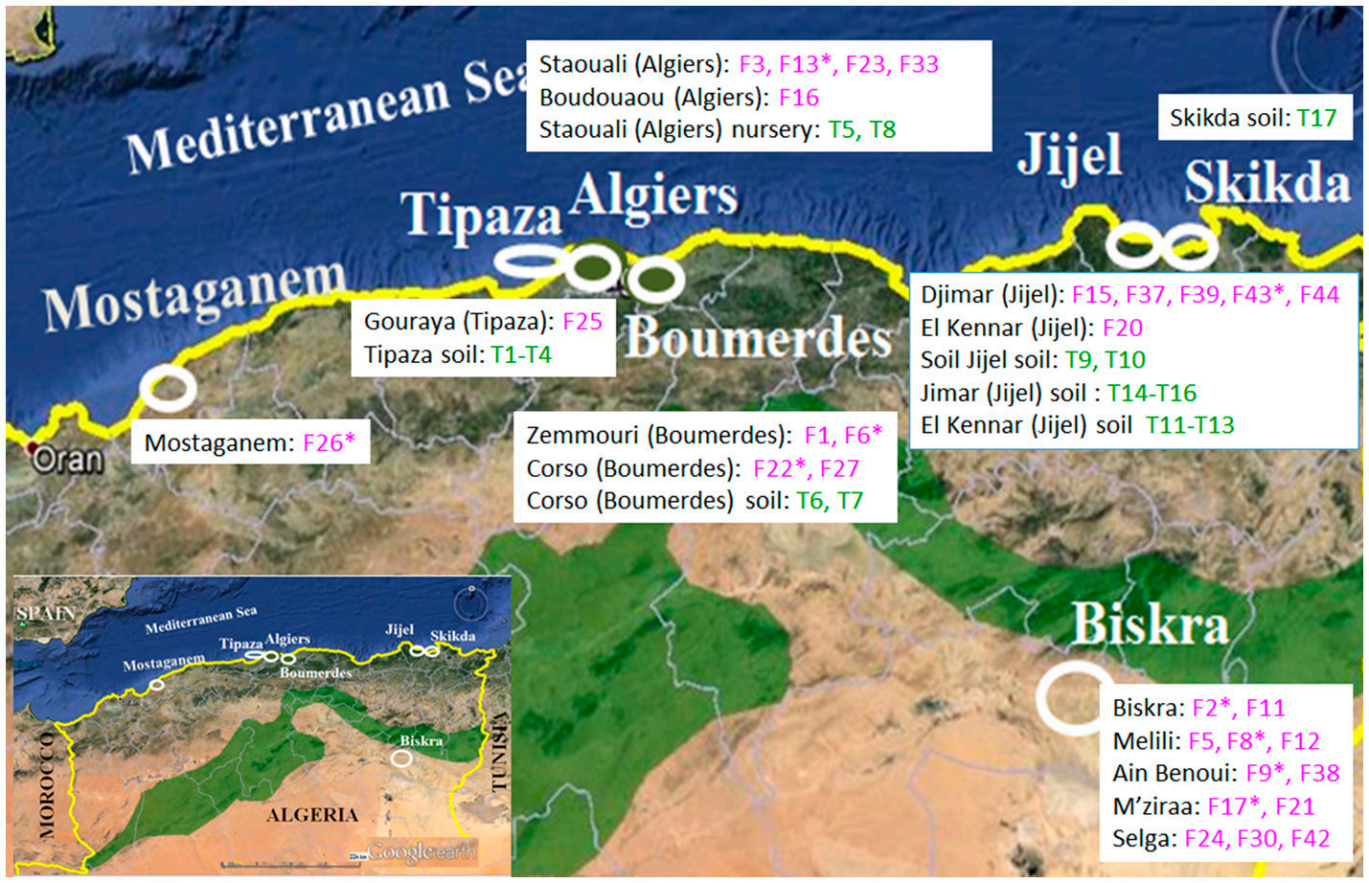
Location of the tomato production fields where the *Fusarium* (F) and *Trichoderma* (T) isolates were collected in Algeria. Illustration source: www.google.com/earth.

Surveys were conducted from 2012 to 2015 in seven tomato growing areas in Algeria: Mostaganem, Tipaza, Algiers, Boumerdes, Skikda, and Jijel, which are located on the coast, and Biskra in the interior of the country (Figure 1). Plants showing typical *Fusarium* disease symptoms (vascular tissue discoloration, leaf wilting or crown, and root rot) were collected. Tomato tissue fragments from diseased plants were surface-disinfected with 2% sodium hypochlorite for 10 min and rinsed thoroughly with sterile distilled water, and plated on potato dextrose agar (PDA, Difco Laboratories, Detroit, USA). Fifty fungal isolates recovered from these seven tomato production areas developed colonies with the typical phenotype of *Fo* (Table 1). Thus, monosporic cultures were obtained and the fungi preserved in the Mycological Collection of the Biotechnology Research Center (CRBt, Constatine, Algeria). In addition, two *Fo* isolates provided by Prof. Díaz-Mínguez (Institute CIALE, Salamanca, Spain) were used as references in this study: Forlc of FORL and 4287 of FOL (Di Pietro and Roncero, 1998). All *Fo* isolates were kept at 4°C on PDA plates.

**Table 1:** Algerian *F. oxysporum* (*Fo*) isolates studied.

**Code**	**Origin**	***SIX1^a^***	**f. sp**	**Symptoms severity on RG*^b^***	**Symptoms severity on SM*^b^***
F1	Zemmouri, Boumerdes	+	FOL	0.0 e	1.3 ± 0.5 e
F2	Biskra	−	FORL	3.6 ± 0.5 a	ND*^d^*
F3	Staouali, Algiers	+	FOL	0.0 e	1.3 ± 0.5 e
F5	Melili, Biskra	+	FOL	0.0 e	2.0 ± 0.7 bcde
F6	Zemmouri, Boumerdes	−	FORL	3.5 ± 0.7 a	ND
F8	Melili, Biskra	−	FORL	1.3 ± 0.5 d	ND
F9	Ain Benoui, Biskra	−	FORL	1.3 ± 0.5 d	ND
F11	Biskra	+	FOL	0.0 e	3.1 ± 0.6 ab
F12	Melili, Biskra	+	FOL	0.0 e	2.8 ± 0.9 abc
F13	Staouali, Algiers	−	FORL	1.2 ± 0.5 d	ND
F15	Djimar, Jijel	+	FOL	0.0 e	3.6 ± 0.5 a
F16	Boudouaou, Algiers	+	FOL	0.0 e	1.4 ± 0.5 e
F17	M'ziraa, Biskra	−	FORL	2.5 ± 0.5 bc	ND
F20	El Kennar, Jijel	+	FOL	0.0 e	1.3 ± 0.5 e
F21	M'ziraa, Biskra	+	FOL	0.0 e	1.3 ± 0.5 e
F22	Corso, Boumerdes	−	FORL	1.6 ± 0.5 cd	ND
F23	Staouali, Algiers	+	FOL	0.0 e	1.6 ± 0.5 cde
F24	Selga, Biskra	+	FOL	0.0 e	1.3 ± 0.5 e
F25	Gouraya, Tipaza	+	FOL	0.0 e	2.0 ± 0.5 bcde
F26	Mostagenem	−	FORL	1.9 ± 0.3 cd	ND
F27	Corso, Boumerdes	+	FOL	0.0 e	1.3 ± 0.5 e
F30	Selga, Biskra	+	FOL	0.0 e	3.7 ± 0.5 a
F33	Staouali, Algiers	+	FOL	0.0 e	2.1 ± 0.6 bcde
F37	Djimar, Jijel	+	FOL	0.0 e	2.7 ± 0.7 abcd
F38	Ain Bennoui, Biskra	+	FOL	0.0 e	1.3 ± 0.5 e
F39	Djimar, Jijel	+	FOL	0.0 e	1.5 ± 0.5 e
F42	Selga, Biskra	+	FOL	0.0 e	2.5 ± 0.5 abcde
F43	Djimar, Jijel	−	FORL	1.9 ± 0.3 cd	ND
F44	Djimar, Jijel	−	*Fo*	0.0 e	0.0 f
4287*^c^*	Córdoba, Spain	+	FOL	0.0 e	2.8 ± 0.4 abc
Forlc*^c^*	Gaza	−	FORL	3.4 ± 0.7 ab	ND

*Information corresponding to the code, isolation location, isolate classification as ff. spp. lycopersici (FOL) or radicis lycopersici (FORL) based on the amplification of the SIX1 gene, and the severity of symptoms developed on tomato “Rio Grande” (RG) and/or “Super Marmande” (SM) varieties for all 29 Fo isolates collected from diseased tomato plants in Algeria*.

a *Presence (+) or absence (−) of 992 bp PCR fragment using the primers P12-F2R and P12-R1 on the target gene SIX1*.

b *Ten plants were used to test each Fo isolate, 4 weeks after inoculation in 14-day-old tomato plants, and the symptom severity was registered using a scale of 0–4. For each column, the values (mean±SD) with different letters denote the statistical significance determined by ANOVA followed by Tukey test (P < 0.05)*.

c *Isolates 4287 and Forlc were included as references of FOL and FOLR, respectively*.

d *ND, not determined*.

Soil samples were taken in tomato crop fields with a low incidence of Fusarium wilt from four Algerian littoral areas (Tipaza, Boumerdes, Jijel, and Skika), and nursery tomato plants samples were also collected from the greenhouses at the Industrial and Vegetable Crops Institute (ITCMI, Staouali, Algiers). The soil samples were taken at a depth of 10 cm at each site, placed in sterile polyethylene bags and transported to the laboratory. Ten grams of soil from each sample were mixed with 90 mL of sterile water by magnetic shaking for 1 h. An aliquot of each sample was sown on *Trichoderma*-selective medium (Elad et al., 1981) and then incubated at 25°C for 5 days. In the case of the nursery tomato plants, roots were disinfected with 2% sodium hypochlorite and washed with sterile water and plated on PDA medium containing 50 mg/L streptomycin. Monoconidial cultures of *Trichoderma* spp. isolated from the soil and plant samples were obtained.

### Plant material

Two susceptible commercial tomato cultivars were used in the pathogenicity tests of the *Fo* isolates (Hibar et al., 2005): “Super Marmande” (SM) and “Rio Grande” (RG) (Thompson & Morgan, Ipswich, UK) for FOL and FORL, respectively. Tomato seeds were superficially disinfected as previously described (Rubio et al., 2017b).

### Genomic DNA extraction

Genomic DNA from *Fusarium* and *Trichoderma* isolates was extracted after growing in potato dextrose broth (PDB, Difco) medium at 25°C and 120 rpm for 5 and 2 days, respectively. Fungal mycelia for DNA extraction were obtained as previously described (Hermosa et al., 2004). Fungal genomic DNA was isolated following the protocol described in Lee et al. (1988).

### PCR reactions with *Fusarium* DNA

#### Identification of FOL races and FORL

In order to support the identification based on morphological characteristics of the 50 *Fusarium* isolates obtained from diseased tomato plants, a PCR reaction using primers PFO2 and PFO3 (Table 2), previously described as specific for *Fo* (Edel et al., 2000), was carried out. A total volume of 25 μL containing 20 ng of *Fo* genomic DNA, 10 pmol of each of the primers indicated above and one unit of *Taq* DNA polymerase (Biotools B&M Lab., Madrid, Spain) was used to perform the PCR. The amplification program included an initial denaturalization cycle of 5 min at 95°C, followed by 30 cycles of 30 s at 95°C, 30 s at 62°C, 30 s at 72°C, and a final extension step of 3 min at 72°C in a Applied Biosystems GeneAmp PCR System 9700 thermal cycler. Following amplification, the PCR products were observed after electrophoresis on 2% agarose gels.

**Table 2:** Primers used in this study and size of amplicons.

**Primer**	**Sequence (5′-3′)**	**Amplicon (in bp)**	**Target**	**References**
PFO2	CCCAGGGTATTACACGGT	70	rDNA-*Fo*	Edel et al., 2000
PFO3	CGGGGGATAAAGGCGG			Edel et al., 2000
P12-F2B	TATCCCTCCGGATTTTGAGC	992	*SIX1*	van der Does et al., 2008
P12-R1	AATAGAGCCTGCAAAGCATG			Rep et al., 2004
SIX3-F1	CCAGCCAGAAGGCCAGTTT	608	*SIX3*	van der Does et al., 2008
SIX3-R2	GGCAATTAACCACTCTGCC			van der Does et al., 2008
SIX4-F1	TCAGGCTTCACTTAGCATAC	967	*SIX4*	van der Does et al., 2008
SIX4-R1	GCCGACCGAAAAACCCTAA			van der Does et al., 2008
SIX3-G121AF2*^a^*	ACGGGGTAACCCATATTGCA	429	*SIX3*	Lievens et al., 2009
SIX3-G134AF2*^a^*	TTGCGTGTTTCCCGGCCA	414	*SIX3*	Lievens et al., 2009
SIX3-G137CF2*^a^*	GCGTGTTTCCCGGCCGCCC	412	*SIX3*	Lievens et al., 2009
ITS1*^b^*	AATAGAGCCTGCAAAGCATG	580–600	ITS	White et al., 1990
ITS4 *^b^*	GCCATCCTTGGAGACCAGC			White et al., 1990
EF1-728F*^b^*	GCCATCCTTGGAGACCAGC	520–565	*tef1alpha*	Chaverri et al., 2003
tef1-rev *^b^*	GCCATCCTTGGAGACCAGC			Kullnig-Gradinger et al., 2002

a *When used with SIX3-R2, a fragment of 429, 414 or 412 bp is generated for FOL race 3 isolates by PCR*.

b *Primer pairs used to amplify fragments and sequencing from Trichoderma isolates*.

All *Fo* isolates were subjected to PCR analysis using primer sets P12-F2B/P12-R1 (*SIX1* gene), SIX3-F1/SIX3-R2, SIX4-F1/SIX4-R1, SIX3-G121A-F2/SIX3-R2, SIX3-G134A-F2/SIX3-R2, and/or SIX3-G137C-F1/SIX3-R2 (Table 2). Reaction mixtures and Thermal conditions were as previously described (van der Does et al., 2008; Lievens et al., 2009). PCR products were electrophoresed as described above, and the results of this PCR analysis were scored as previously described (Lievens et al., 2009).

#### Inter simple sequence repeat (ISSR) PCR analysis

The genetic diversity existing among 31 *Fo* isolates (the 29 isolated in Algeria and the two Spanish included as references) was analyzed by PCR with the primers (GA)_9_C and (GA)_9_T and the *Taq* polymerase system (Biotools). The PCR amplification was carried out in a 25 μL reaction volume with 2.5 μL of 10X PCR buffer, 0.2 mM dNTPs, 10 pmol of primer, 1.25 units of *Taq* DNA polymerase and 50 ng genomic DNA as the template. For each primer-isolate combination, amplifications were repeated two times to assure reproducibility. The PCR program was as previously described (Nirmaladevi et al., 2016). PCR products were electrophoresed on 1.5% agarose gel as described above, and photographs were taken when visualized in a UV transilluminator.

For data analysis, the presence or absence of a given band was scored as 1 and 0, respectively. The application of the Jaccard's coefficient to the binary matrix served to calculate the pairwise distance among the isolates, which were clustered by UPGMA (Unweighted Pair Group method using arithmetic means). Genetic diversity for each primer was calculated as previously described (Owen et al., 1998) using the following formula (*H* = 1–*Σxi*^2^), where H is the expected heterozygosity and *xi* is the frequency of allele *i*, including “nulls” as a separate band.

### PCR amplification, sequencing and DNA analysis for *Trichoderma* isolates

The amplification and sequencing of the ITS regions and a fragment of ca. 0.56 kb of the *tef1*α (translation elongation factor 1-alpha) gene from 17 *Trichoderma* isolates were performed using the primers ITS1/ITS4 and EF1-728F/tef1-rev (Table 2), respectively, as described previously (Hermosa et al., 2004; Sadfi-Zouaoui et al., 2009). The *Trichoderma* ITS and *tef1*α sequences obtained in this work were analyzed by comparison with sequences of *Trichoderma* strains deposited in the NCBI GenBank database.

### Pathogenicity test for *Fo* isolates

Tomato seeds of “SM” and “RG” varieties were disinfected as indicated above, and sowed in a commercial substrate Projar Professional-Comercial (Projar SA, Valencia, Spain), previously autoclaved for 1 h at 121°C on two successive days and deposited in multi-cell growing trays. Seedlings were maintained under greenhouse conditions and inoculated with *Fo* when they were 14 days old as previously described (Rubio et al., 2017b), except that 3 L pots containing a sterilized mixture of sand:peat:vermiculite (1:1:1) were used in the present study. Five pots per condition and two seedlings per pot were used for each *Fo*. Results were expressed for “SM” and “RG” as symptoms severity index, using the following symptoms scale (0–4): 0, healthy plant; 1, 2, and 3, slight, moderate and severe chlorosis, wilting or stunting “SM” plant, respectively; 1, 2, and 3, slight, moderate and severe dark brown lesions on crown and roots of “RG” plant, respectively; and 4, dead plant.

### *In vitro* antifungal assays

Isolates T5, T8, T9, and T17 of *Trichoderma* were *in vitro* tested for their antifungal potential against the *Fo* isolates F17, Forlc, 4287, and F42 using direct confrontation and membrane assays as previously described (Rubio et al., 2009, 2017b), except that the *Fo* colony diameters were measured after 6 and 5 days, respectively. Each *Trichoderma*-*Fo* combination was tested in triplicate. Results were expressed as the percentage of growth inhibition of each *Fo* by each *Trichoderma* isolate with respect to the mean colony diameters of each *Fo* grown alone (direct confrontation assay) or *Fo* control culture (membrane assays).

### *In vivo* biocontrol assays in tomato plants

The ability of three *Trichoderma* isolates (T8, T9, and T17) to control diseases caused by three *Fo* isolates (Forlc, 4287, and F42) in tomato cv. “SM” was evaluated in *in vivo* assays. Surface-sterilized tomato seeds were coated with *Trichoderma* conidia (1 mL of an aqueous suspension containing 2 × 10^7^ conidia per mL was used to coat 30 seeds) as previously described (Pérez et al., 2015). *Trichoderma*-treated tomato seeds were sown in the commercial substrate indicated above in multi-cell growing trays maintained in a greenhouse. Untreated tomato seeds were used as control. *Fo* inoculation was performed as previously described (Rubio et al., 2017b). Ten pots containing one seedling per pot were used in each *Trichoderma*-*Fo* combination tested. The experiment lasted 3 weeks and was conducted twice with similar results. The severity of the symptoms was recorded using the index range (0–4) already mentioned. The percentage of the incidence of disease (ID) and the decrease in ID (DID) due to the treatment with *Trichoderma* were determined as previously described (Song et al., 2004).

### Statistical analysis

Data from *in vitro* and *in vivo* assays were subjected to analysis of variance (ANOVA) using STATISTIC v10.0 (NH Analytical Software, Roseville, USA) and means compared by Tukey's test (*P* < 0.05).

## Results

### Identification of algerian *Fo* isolates

The amplification of a 70 bp PCR fragment with the primer pair PFO2–PFO3 served to identify 29 out of 50 fungal isolates collected from diseased tomato plants in Algeria as *Fo* (Table 1, Figure 1). In order to discriminate between FORL and FOL isolates, PCR amplifications of the *SIX1* gene, which encodes a FOL virulence factor toward tomato, were performed using the P12-F2R and P12-R1 primers, and the DNA from two reference isolates were also included as controls. As a result, 20 isolates, including the Spanish isolate 4287 used as a FOL reference, exhibited the 992-bp PCR fragment indicative of the f. sp. FOL (Table 1).

All of the 31 (29 from Algeria and two control) isolates of *Fo* were utilized in the pathogenicity tests performed on tomato plants with the aim to confirm the identification of each isolate as being either FOL and FORL as shown by the *SIX1* PCR amplifications. The results of pathogenicity tests are presented in Table 1. Ten (nine collected in Algeria and the reference isolate Forlc) out of 11 FORL isolates presented virulence on tomato cv. “RG”. Plants inoculated with isolates F2 and F6 gave the highest symptom severity indexes, these values being significantly different from those obtained for the seven other Algerian FORL isolates, although no differences were detected with that of Forlc. Then, a pathogenicity test was performed on tomato plants cv. “SM” for the remaining 20 FOL isolates, plus the *Fo* isolate F44, that were non-virulent on “RG.” All FOL isolates (19 from Algeria and the reference isolate 4287) caused the typical symptoms of wilt disease, which were observed 4 weeks after the plants were inoculated. The symptoms severity values obtained for FOL-inoculated plants were diverse, ranging from 1.3 (for seven isolates) to 3.7 for F30. Isolates F15 and F30 gave the highest values but did not differ significantly from the values recorded for the plants inoculated with isolate 4287. Since the *Fo* isolate F44 did not cause disease symptoms on either “RG” or “SM” tomato plants, it was recorded as being a tomato non-pathogenic *Fo* isolate.

Additional PCR amplifications were carried out with *SIX3* and *SIX4* pair primers (Table 2) to discriminate the FOL races. The three different amplification patterns obtained with the 31 *Fo* isolates showed the presence of FOL races 2 and 3 and FORL among the Algerian ones (Table 3). A 608-bp fragment of *SIX3* was amplified with SIX3-F1 and SIX3-R2 primers for 20 isolates, but no amplification was observed with SIX4-F1 and SIX4-R1 primers, indicating that they were not FOL race 1. This result is in concordance with the amplification of the 992-bp PCR fragment of the *SIX1* gene seen in these 20 isolates, and supports the results obtained from the pathogenicity test. The presence of the 412-bp fragment PCR detected for isolates F12 and F20 with the SIX3-G137C-F1 and SIX3-R2 pair primers, and the absence of an amplicon when SIX3-G121AF2 or SIX3-G134AF2 primer was combined with the primer SIX3-R2, indicated that these isolates were FOL race 3. No amplification bands for genes *SIX3* and *SIX4* were observed for the 10 isolates, including the FORL reference isolate, scored previously as *SIX1* negative and exhibiting virulence on tomato cv. “RG”, confirming that they were FORL.

**Table 3:** Molecular identification of Algerian *F. oxysporum* (*Fo*) isolates based on patterns of *SIX3* and *SIX4* gene amplifications (+, amplicon was present; −, amplicon was absent).

**Isolates**	**Primer pairs**			***Fo* identify**
	**SIX3-F1/SIX3-R2**	**SIX4-F1/SIX4-R1**	**SIX3-G137C-F1/SIX3-R2**	
	**(608 bp)**	**(967 bp)**	**(412 bp)**	
F1, F3, F5, F11, F15, F16, F21, F23-F25, F27, F30, F33, F37-F39, F42, 4287*^a^*	+	−	−	Race 2 FOL
F12, F20	+	−	+	Race 3 FOL
F2, F6, F8, F9, F13, F17, F22, F26, F43, F44				
Forlc*^b^*	−	−	−	FORL

a *Isolate 4287 was included as reference for f. sp. lycopersici (FOL) race 2*.

b *Isolate Forlc was included as reference for f. sp. radicis lycopersici (FORL)*.

### Genetic diversity among algerian *Fo* isolates by ISSR fingerprinting

Two primers were analyzed by their capacity to produce polymorphism amplicons on the set of 31 *Fo* isolates, which included the 29 from Algeria and the reference isolates Forlc and 4287. The (GC)_9_C or (GT)_9_T primers resulted in reproducible banding patterns for 26 out of 31 isolates (Table 4). The two primers (GA)_9_C and (GA)_9_T produced 20 and 17 bands among 22 and 26 *Fo* isolates, respectively. The (GA)_9_C primer produced 1–8 bands ranging from approximately 400–1,900 bp and (GA)_9_T produced 1–7 bands ranging from 300 to 2,000 bp. The average Nei's gene diversity was 0.22, with the highest value recorded for primer (GA)_9_T (0.26).

**Table 4:** ISSR typing using two primers to analyze the genetic diversity among 31 isolates of *F. oxysporum*, 29 collected in Algeria and 4287 and Folrc included as references, with the Nei's gene diversity (h).

**ISSR primer**	**Number of loci**	**Number of isolates with null band**	**Nei's gene diversity (h)**
(GA)_9_C	20	9	0.19
(GA)_9_T	17	5	0.26

**Figure 2:**
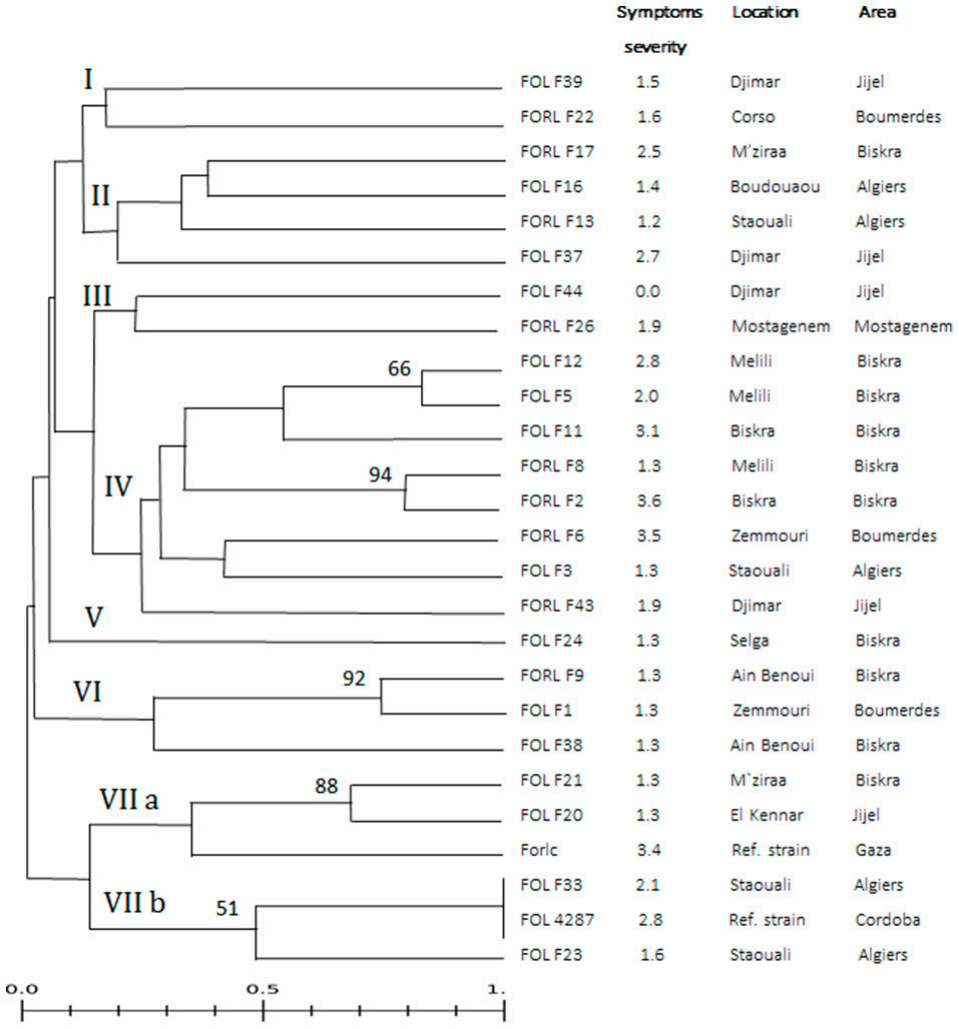
Grouping of Algerian *F. oxysporum* (Fo) isolates based on genetic similarity. Unweighted pair-group method algorithm dendrogram of relative genetic similarity among 26 *Fo* isolates, calculated in the ISSR analysis with (GA)_9_C and (GA)_9_T primers and using Jaccard's coefficient. Relative support for particular clades in the UPGMA dendrogram was estimated by using 1,000 replications of the bootstrap procedure.

The drawn UPGMA dendrogram (Figure 2), based on the band patterns observed with both primers and the Jaccard similarity coefficient, showed high diversity among the 26 *Fo* isolates. A similarity index of 1 was only recorded for the FOL race 2 isolate 4287 and FOL isolate F33. The typology of this dendrogram clustered the 26 *Fo* isolates into seven major groups, identified as I-VII, using a similarity index limit of 0.26. In general, FOL and FORL isolates were distributed across the dendrogram, and their clusters did not show a correlation with either the severity of the symptoms found in the pathogenicity tests or the geographical locations in which the isolates were collected. Nevertheless, five out of 10 isolates collected in the Biskra area were located within group IV, but this cluster also included four out of the 10 FORL isolates and it was not supported with bootstrap stability.

### Identification of *Trichoderma* isolates and potential biocontrol activity against *Fo*

Seventeen *Trichoderma* isolates were collected, 15 from cultivated tomato soils without symptoms of disease and two of nursery tomato plants (Figure 1). Their identity was confirmed at the species level by analyzing the sequences of two molecular markers (Table 5). The sequences containing the ITS1, ITS2, and 5.8S gene were 584–600 bp in length, and their alignment served to identify three different sequence types. A ca 600 bp fragment of the *tef1*α gene fragment also served to identify three different types of sequences. Both ITS and *tef1*α sequences gave 100% identity with sequences from reference strains deposited in the databases, and identified four isolates as *T. harzianum*, 12 as *T. asperellum* and one as *T. ghanense*.

**Table 5:** Source and identification of Algerian *Trichoderma* isolates analyzed in this study.

**Isolate code**	**Source/geographical origin**	**Identified as**	**100% Identity to strain/accession number*^a^***
T1	Soil/Tipaza	*T. harzianum*	GJS 99-5/FJ442236
T2	Soil/Tipaza	*T. asperellum*	CBS 433.97/AF456907
T3	Soil/Tipaza	*T. asperellum*	CBS 433.97/AF456907
T4	Soil/Tipaza	*T. asperellum*	CBS 433.97/AF456907
T5	ITCMI*^b^* nursery/Staouali Algiers	*T. harzianum*	GJS 99-5/FJ442236
T6	Soil/Corso, Boumerdes	*T. harzianum*	GJS 99-5/FJ442236
T7	Soil/Corso, Boumerdes	*T. harzianum*	GJS 99-5/FJ442236
T8	ITCMI*^b^* nursery/Staouali Algiers	*T. ghanense*	DAOM 220800/EU280100
T9	Soil/Jijel	*T. asperellum*	CBS 433.97/AF456907
T10	Soil/Jijel	*T. asperellum*	CBS 433.97/AF456907
T11	Soil/El Kennar, Jijel	*T. asperellum*	CBS 433.97/AF456907
T12	Soil/El Kennar, Jijel	*T. asperellum*	CBS 433.97/AF456907
T13	Soil/El Kennar, Jijel	*T. asperellum*	CBS 433.97/AF456907
T14	Soil/Jimar, Jijel	*T. asperellum*	CBS 433.97/AF456907
T15	Soil/Djimar, Jijel	*T. asperellum*	CBS 433.97/AF456907
T16	Soil/Djimar, Jijel	*T. asperellum*	CBS 433.97/AF456907
T17	Soil/Skikda	*T. asperellum*	CBS 433.97/AF456907

*The classification of Trichoderma species was based on the sequences obtained from ITS1-ITS4 region of the rDNA and a fragment of tef1alpha gene*.

a *100% identity of ITS and tef1alpha-fragment sequences with database (NCBI GenBank) sequences from reference Trichoderma strains*.

b *ITCMI: Industrial and Vegetable Crops Institute, Staouali Algiers, Algeria. Strains are available for research proposals in the Mycological Collection of the Biotechnology Research Center (CRBt, Constatine, Algeria) (www.crbt.dz)*.

Two Algerian *Fo* isolates, representing FOL and FORL, the reference isolates FOL 4287 and FORL Forlc, and four *Trichoderma* isolates (T5, T8, T9, and T17), representing the species diversity found within the Algerian samples (Figure 1), were included in *in vitro* antifungal assays to test the biocontrol potential of *Trichoderma* against *Fo* (Table 6). When confronted in dual cultures, the four *Trichoderma* isolates were able to overgrow the colonies of the four *Fo* isolates and sporulate. Differences were only detected in the growth inhibition of the dual cultures containing T5 against Forlc or 4287 and T8 against F17.

**Table 6:** Antagonism of Algerian *Trichoderma* spp. isolates against *F. oxysporum* (*Fo*).

***Trichoderma*-*Fo* combination**	**Dual culture**	**Cellophane**	**Dialysis membrane**
T5-FORL F17	64.6 ± 1.1 ab	17.0 ± 1.0 de	47.6 ± 1.4 de
T5-FORL Forlc	69.8 ± 0.0 a	12.5 ± 2.9 e	29.8 ± 2.2 fg
T5-FOL 4287	70.6 ± 1.1 a	35.3 ± 2.1 bcd	27.5 ± 1.0 fg
T5-FOL F42	69.5 ± 0.0 ab	35.1 ± 2.4 bcd	22.6 ± 2.5 g
T8-FORL F17	59.1 ± 1.1 b	35.8 ± 1.3 bcd	57.7 ± 1.5 bcd
T8-FORL Forlc	62.8 ± 0.0 ab	63.5 ± 1.8 a	72.5 ± 1.5 ab
T8-FOL 4287	63.0 ± 1.0 ab	47.1 ± 1.5 abc	53.7 ± 1.0 cd
T8-FOL F42	68.0 ± 1.1 ab	51.2 ± 1.4 ab	56.9 ± 1.3 bcd
T9-FORL F17	62.4 ± 1.5 ab	37.0 ± 1.8 bc	41.8 ± 2.6 def
T9-FORL Forlc	63.6 ± 1.1 ab	62.7 ± 0.6 a	78.5 ± 1.0 a
T9-FOL 4287	63.0 ± 0.0 ab	46.2 ± 1.0 abc	68.3 ± 2.9 abc
T9-FOL F42	66.4 ± 1.1 ab	26.5 ± 1.5 cde	30.5 ± 1.91 efg
T17-F17	64.6 ± 1.1 ab	28.5 ± 1.7 cde	49.2 ± 1.4 d
T17-FORL Forlc	63.6 ± 1.1 ab	64.4 ± 2.2 a	82.1 ± 2.6 a
T17-FOL 4287	63.0 ± 0.0 ab	52.1 ± 1.9 ab	68.9 ± 2.3 abc
T17-FOL F42	68.7 ± 1.1 ab	29.9 ± 1.9 cde	31.3 ± 2.9 efg

*Colony growth inhibition of Fo f. sp radicis lycopersici F17 and Forlc, and Fo f. sp. lycopersici 4287 and F42 isolates by T. harzianum T5, T. ghanense T8 and T. asperellum T9 and T17, after dual culture growth for 6 days and by metabolites from 2-day cultures of Trichoderma strains on cellophane or a 14-kDa cut-off dialysis membrane for 5 days^a^. ^a^Results are expressed as the percentage of inhibition of each Fo isolate by each Trichoderma with respect to the mean colony diameter of each fungus grown alone. Values are means of three replicates with the corresponding standard deviation. Values in the same row with different letters are significantly different according to Tukey's test (P < 0.05)*.

When *Trichoderma* isolates were tested on cellophane and dialysis (cut-off 14 kDa) membranes, the subsequent *Fo* cultivation always led to a reduced colony size. The effects of metabolites and hydrolytic enzymes (cellophane) or only metabolites (dialysis) secreted by the four *Trichoderma* isolates on the growth of the four *Fo* isolates are reported as *Fo* colony growth inhibition percentages in Table 6. No significant differences were found among the four *Trichoderma* isolates against FOL 4287 in the cellophane assay, but differences were observed for the inhibition of FORL F17 between T9 and T5 in that same assay. The isolate T5 also displayed a significantly worse performance against Forlc. In the case of F42, significant growth inhibition differences were detected between the best isolate (T8: 51.2%) and the worst (T9: 26.5%).

In dialysis membrane tests (Table 6), no significant differences were observed in the antifungal activities of the four *Trichoderma* isolates against FORL F17. However, T5 showed the significantly lowest inhibition values against Forlc and 4287. Isolates T8 and T9 also showed the significant highest and lowest inhibition records against FOL F42, respectively. These results indicate that *Fo* is sensitive to metabolites released by T8, T9, and T17 and, at least in *in vitro* assays, these isolates had the highest antagonistic potential against *Fo*. These three *Trichoderma* isolates were selected for succesive *in planta* tests.

### Biocontrol of *Trichoderma* against *Fo*

The effect of treatment with *T. ghanense* T8 and *T. asperellum* T9 and T17 on diseases caused by FOL F42, and the reference isolates FOL 4287 and FORL Forlc, in tomato cv. “SM” is shown in Table 7. No symptoms were detected on the non-inoculated plants and those grown from T8-, T9-, or T17-treated seeds (data not shown). The ID caused by Forlc ranged from 80 to 97.5%, while the percentages of the ID in F42- and 4287-inoculated plants were lower. Furthermore, biocontrol activity against *Fo* in tomato was detected for the three *Trichoderma* strains tested. The highest percentages of DID were recorded for isolate T8, which were 53.1 and 48.3%, against Forlc and F42, respectively. Isolate T9 gave a higher DID against FOL 4287 than T8 and T17. Based on these results, *T. ghanense* T8 seemed to be the best isolate for controlling tomato diseases caused by FORL and FOL.

**Table 7:** Incidence of the disease (ID) caused by *F. oxysporum* f. sp. *radicis lycopersici* Forlc and *F. oxysporum* f. sp. *lycopersici* F42 and 4287 in “Super Marmande” tomato plants single treated with *T. ghanense* T8 and *T. asperellum* T9 and T17*^a^*.

***Fusarium* treatments**	**ID (%)**	***Trichoderma-Fusarium* treatments**	**ID (%)**	**Decrease of disease incidence (DID)*^b^***
FORL Forlc	80 ± 10.5	T8-Forlc	37.5 ± 13.2 a	53.1
FORL Forlc	97.5 ± 7.9	T9-Forlc	77.5 ± 7.9 b	20.5
Forlc	90 ± 12.9	T17-Forlc	80 ± 10.5 b	11.1
FOL 4287	77.5 ± 7.9	T8-4287	57.5 ± 12.1 ab	25.8
FOL 4287	62.5 ± 13.2	T9-4287	37.5 ± 13.2 a	40.0
FOL 4287	82.5 ± 12.1	T17-4287	67.5 ± 12.1 b	18.2
FOL F42	72.5 ± 7.9	T8-F42	37.5 ± 13.2 a	48.3
FOL F42	55 ± 10.5	T9-F42	30 ± 10.5 a	45.4
FOL F42	42.5 ± 12.1	T17-F42	30 ± 10.5 a	29.4

a *Plants were obtained from untreated or Trichoderma-treated seeds. Fusarium isolates were applied when tomato seedlings were 14 days old, and the incidence of disease was evaluated 3 weeks after Fusarium inoculation. Data are the mean of the results obtained from 10 plants per single Fusarium or combined Trichoderma-Fusarium treatments with the corresponding standard deviation. For each Trichoderma-Fo combination tested, ID values followed by different letters are significantly different according to Tukey's test (P < 0.05)*.

b *DID was calculated as the difference between DI in the Fo treatment and in the Fo-Trichoderma treatment divided by DI in the Fo treatment expressed as a percentage*.

## Discussion

The Mediterranean climate with warm to hot, dry and long summers and very mild winters favors tomato crop field production, which can be accompanied by soil-borne diseases, as is the case of those produced by *Fo* (Muñoz et al., 2008). The use of cultivars resistant to FOL and grafted material to prevent FORL (only tolerant varieties are available against this last f. sp.) are common strategies employed in many countries that have led to a decrease in *Fusarium* diseases in tomato crops (Hibar et al., 2007; Baysal et al., 2009). However, *Fo* diseases continue to be a major sanitary problem in tomato production areas of countries like Turkey, Malta, Morocco, Tunisia, Algeria (Porta-Puglia and Mifsud, 2005; Baysal et al., 2009; Edel-Hermann et al., 2012; Taghdi et al., 2015), or India (Nirmaladevi et al., 2016). In many cases, in addition to the lack of knowledge concerning the pathogenic forms present in a given cultivation area, farmers produce their own seeds from old tomato cultivars, which can lead to the spread of FOL and FORL pathogens through seeds, transplants and contaminated soil (Edel-Hermann et al., 2012).

Nineteen out of the 29 Algerian isolates of *Fo* analyzed in our study, collected between 2012 and 2015, have been classified as FOL, according to the pathogenicity tests and the amplification of the *SIX* genes. Previous studies have highlighted the usefulness of primers designed to *SIX* genes for the rapid discrimination by PCR-based methods of FOL races among the *Fusarium* species (van der Does et al., 2008; Lievens et al., 2009). Our results indicate that *SIX* genes were useful to discriminate between FOL and FORL isolates, since the results obtained from the amplification of the *SIX* genes and the pathogenicity tests showed a high level of concordance in 30 out of the 31 *Fo* isolates analyzed. Isolate F44 did not cause symptoms when used to inoculate the “RG” tomato variety. Moreover, it is not uncommon to find non-pathogenic *Fo* strains among isolates originally obtained from collections (Cai et al., 2003), monosporic-origin inocula (Boix-Ruíz et al., 2015), or soil samples (Jelinski et al., 2017). FOL race 2 was overrepresented among the Algerian isolates tested, although our results show that race 3 (isolates F12 and F20) is also present in this country. Our results agree with those reported for *Fo* isolates collected from the Mediterranean coast of Turkey (Baysal et al., 2009), where race 2 and 3 of FOL, and FORL were detected. While FOL race 3 was overrepresented in Turkish samples, in the present study its prevalence only reached 6.9%. The existence of race 3 in a North African country such as Algeria reinforces the fact that this FOL race is distributed worldwide.

Since the use of grafted material is not a common practice in this North African region, it could be expected that FORL is often present in tomato growing areas (Hibar et al., 2007; Edel-Hermann et al., 2012). Nine out of the 29 Algerian isolates analyzed in our study were classified as FORL, representing only 34.5% of total number of isolates. A previous study carried out with *Fo* isolates from eight Mediterranean countries, showed that the Algerian isolates, all of them identified as FORL, were genetically diverse from those of the other countries (Edel-Hermann et al., 2012). Our results show the presence of FORL and FOL, and even different FOL races in a same location, i.e., isolates F5, F8, and F12 in Melili (Biskra). Thus, the 19 Algerian isolates identified as FOL in the present study represent the first formal report of the presence of FOL race 2 and 3 in Algeria.

Correlation between FOL races and field location has been proposed for isolates sampled in Turkey (Baysal et al., 2009). For instance, race 3 and FORL were associated with coastal fields, whereas race 2 was more frequent in inland crops. However, our study, covering the seven major tomato production areas in Algeria and including isolates from 13 geographical locations, did not identify a predictable dissemination pattern for *Fo* (Figure 1). FORL and the races 2 and 3 of FOL were detected in inland fields and at a single tomato growing area in Melili (Biskra). In addition, seven out of the 17 FOL race 2 isolates were also sampled in the Biskra region.

Molecular markers based on PCR, such as ISSR and RAPD, are useful to analyze genetic variation within and between fungal species. Several studies have used the ISSR analysis to assess genetic variability in *Fo* from different food crops (Dubey and Shio, 2008; Baysal et al., 2009, 2010; Edel-Hermann et al., 2012; Nirmaladevi et al., 2016). Because of the use of longer primers, normally 18–24 bp long, ISSR markers are referred as more reproducible than those generated by RAPD ones (Godwin et al., 1997). In this work, we have examined the genetic diversity among 29 Algerian *Fo* isolates by ISSR analysis, using two primers and including two reference isolates for comparative purposes. Despite the fact that five isolates did not produce amplified bands, a high divergence among isolates was still observed (83.9%). In addition, the polymorphism detected as a whole with these two primers suggests a large genetic variation within FOL and FORL. This is in accordance with the high degree of genetic diversity detected among Indian FOL isolates using the same primers to carry out the ISSR analysis (Nirmaladevi et al., 2016). By contrast, low genetic diversity was reported for race 2 isolates in Taiwan, which was the predominant race in that particular study (Sheu and Wang, 2006). RAPD-based genetic diversity within and among VCGs of FORL has also been described (Balmas et al., 2005). Moreover, it has been reported that European *Fo* isolates are less diverse than those from North Africa, and that FORL populations in Algeria, which comprised numerous IGS types and VCGs, presented differences when compared to other populations originating from other Mediterranean countries (Edel-Hermann et al., 2012). This appreciation is supported by the UPGMA tree generated in our study, where most of the Algerian isolates were separately clustered from the two reference isolates of FOL and FORL.

In our ISSR analysis, 26 *Fo* isolates were distributed in seven UPGMA groups, but this clustering did not correspond to any of the field sampling locations, ff. spp. or the level of symptoms of disease in tomato. For instance, as indicated above, FOL races 2 and 3 and FORL occurred in the same location. Some studies have reported correlation between the groupings of *Fo* isolates and geographical origins at various levels, while others have not. Baysal et al. (2009) reported an association between UPGMA groupings and climatic conditions for FOL race 3 isolates coming from Turkey. However, occurrences of more than one race of the *Fo* f. sp. *ciceris* in the same place of India have also been reported (Dubey and Shio, 2008).

*Fo* diseases have become a major threat to tomato production in Algeria because resistant hosts are not always available or used, the chemical treatments usually fail to control FOL and FORL in the field and, as indicated above, farmers themselves are contributing to the spread of these pathogens (Baysal et al., 2009). In addition, it has been proposed that “new pathogenic forms of FORL could have evolved from non-pathogenic local populations in Algeria” (Edel-Hermann et al., 2012). Previous studies have demonstrated the ability of biocontrol strains of *Trichoderma* against Fusarium wilt in different crops, such as tomato (Cotxarrera et al., 2002; Taghdi et al., 2015) and melon (Martínez-Medina et al., 2014). Recently, it has been reported that two *T. harzianum* isolates collected in Western Algeria displayed biocontrol activity against the tomato crown and root rot disease caused by Algerian FORL isolates (Kerroum et al., 2015). It is well established that biocontrol agents isolated from a given crop would have better adaptation to that crop, providing better control than those isolated from other plants (Cook, 1993). Considering the high genetic variability of the FORL and FOL isolates sampled in the different Algerian regions, we performed a survey in nursery tomato plants and tomato field soils with low incidence of *Fusarium* diseases to collect native *Trichoderma* isolates following a similar approach to that used by Taghdi et al. (2015) in Morocco. The most frequently isolated species in our study was *T. asperellum*. This finding agrees with previous studies that described the presence of this species in tomato cultivation fields in other North African countries like Egypt (El Komy et al., 2015) and Morocco (Taghdi et al., 2015). *T. ghanense*, a member of the *Trichoderma* section *Longibrachiatum*, has a worldwide distribution (Druzhinina et al., 2012) and has scarcely been cited as being a biocontrol agent. However, in addition to having plant growth promoting abilities, some *T. ghanense* isolates have shown capacity to control Fusarium wilt in melon (Martínez-Medina et al., 2014) and the root disease caused by *Pythium arrhenomanes* in rice plants (Banaay et al., 2012). In our study, an isolate sampled from a tomato plant from the ITCMI nursery (Staouali, Algiers) was identified as *T. ghanense*.

It is clear that the screening procedures for the selection of biological control agents must include *in vitro* and *in vivo* tests because of the influence of environmental and edaphic factors in the mechanisms of action displayed by the antagonist. Based on the species diversity and abundance of the *Trichoderma* isolates found in Algeria, the isolates T5, T8, T9, and T17 were selected to carry out laboratory tests against two FORL and two FOL isolates in order to explore any antagonistic activity. Results show that the *Trichoderma* isolates T5, T8, T9, and T17 were effective against FORL and FOL via different mechanisms. All of them were able to overgrow FORL and FOL isolates and inhibit their growth. We have previously reported that *T. asperellum* isolates sampled in Morocco were not able to overgrow the colonies of FOL (Taghdi et al., 2015). Thus, the ability to do so, as observed in T9 and T17 against FOL and FORL, must not to be considered a common trait of *T. asperellum* but rather a particular feature of these two isolates. Antibiosis is a major biocontrol mechanism in *Trichoderma* that has been proven to be efficient against FORL and FOL, as indicated by the results obtained in membrane assays. The lowest inhibition values, obtained for most of the T5-*Fo* combinations in the two different membrane assays, are indicate that *T. harzianum* T5 has a lower antifungal activity against FORL and FOL isolates. However, higher *Fo* growth inhibition values in the dialysis membrane assay for eight out of the 16 *Trichoderma*-*Fo* combinations tested was detected. These results show that both FORL and FOL are sensitive to the small compounds secreted by *Trichoderma*. This is in agreement with previous studies showing that metabolites secreted by *Trichoderma* spp. are a major contributor to biocontrol of this fungus against different plant pathogenic fungi (Lorito et al., 2010; Taghdi et al., 2015). No differences were noted in the biocontrol potential of T8, T9, and T17 when representative FORL and FOL isolates were used as targets. Therefore, these three *Trichoderma* isolates were further tested to assess their efficiency suppressing crown and root rot and Fusarium wilt in tomato caused by isolates of FORL and FOL, respectively.

Greenhouse assays performed on susceptible “SM” plants with three *Fo* isolates, representing FORL and FOL and the two reference isolates, showed high ID values in *Trichoderma* untreated plants. The highest reduction in ID corresponded to *T. ghanense* T8 and *T. asperellum* T9, indicating these species could satisfactorily protect tomato against FORL and FOL. The differences in the biocontrol potential observed between *T. asperellum* T9 and T17 show how two isolates of the same species can display similar antagonistic activity against different *Fo* isolates in *in vitro* assays but show different biocontrol behavior in *in planta* tests.

In conclusion, the results of the present study have shown that FORL and races 2 and 3 of FOL are present in Algeria, and that they show high genetic diversity. Also, the results support the hypothesis that native species display better biocontrol, where two isolates of *T. ghanense* and *T. asperellum* can protect against crown and root rot and Fusarium wilt in tomato, in cases of severe disease. Therefore, it can be said that *Trichoderma* biocontrol is a suitable means for the integrated management of these two widespread tomato diseases when used in combination with resistant host plants.

## Author contributions

AD and HB carried out the collection of plant and soil samples and fungal isolation. AD carried out PCR analysis and *in vitro* and greenhouse assays. RH prepared tables and figures. RH, EM, and AD wrote the manuscript. RH designed and led the study. All authors have read and approved the final manuscript.

### Conflict of interest statement

The authors declare that the research was conducted in the absence of any commercial or financial relationships that could be construed as a potential conflict of interest.
